# Evolution and epidemic spread of SARS-CoV-2 in Brazil

**DOI:** 10.1126/science.abd2161

**Published:** 2020-07-23

**Authors:** Darlan S. Candido, Ingra M. Claro, Jaqueline G. de Jesus, William M. Souza, Filipe R. R. Moreira, Simon Dellicour, Thomas A. Mellan, Louis du Plessis, Rafael H. M. Pereira, Flavia C. S. Sales, Erika R. Manuli, Julien Thézé, Luiz Almeida, Mariane T. Menezes, Carolina M. Voloch, Marcilio J. Fumagalli, Thaís M. Coletti, Camila A. M. da Silva, Mariana S. Ramundo, Mariene R. Amorim, Henrique H. Hoeltgebaum, Swapnil Mishra, Mandev S. Gill, Luiz M. Carvalho, Lewis F. Buss, Carlos A. Prete, Jordan Ashworth, Helder I. Nakaya, Pedro S. Peixoto, Oliver J. Brady, Samuel M. Nicholls, Amilcar Tanuri, Átila D. Rossi, Carlos K.V. Braga, Alexandra L. Gerber, Ana Paula de C. Guimarães, Nelson Gaburo, Cecila Salete Alencar, Alessandro C.S. Ferreira, Cristiano X. Lima, José Eduardo Levi, Celso Granato, Giulia M. Ferreira, Ronaldo S. Francisco, Fabiana Granja, Marcia T. Garcia, Maria Luiza Moretti, Mauricio W. Perroud, Terezinha M. P. P. Castiñeiras, Carolina S. Lazari, Sarah C. Hill, Andreza Aruska de Souza Santos, Camila L. Simeoni, Julia Forato, Andrei C. Sposito, Angelica Z. Schreiber, Magnun N. N. Santos, Camila Zolini de Sá, Renan P. Souza, Luciana C. Resende-Moreira, Mauro M. Teixeira, Josy Hubner, Patricia A. F. Leme, Rennan G Moreira, Maurício L. Nogueira, Brazil-UK Centre for Arbovirus Discovery, Diagnosis, Genomics and Epidemiology (CADDE) Genomic Network, Neil M Ferguson, Silvia F. Costa, José Luiz Proenca-Modena, Ana Tereza R. Vasconcelos, Samir Bhatt, Philippe Lemey, Chieh-Hsi Wu, Andrew Rambaut, Nick J. Loman, Renato S. Aguiar, Oliver G. Pybus, Ester C. Sabino, Nuno Rodrigues Faria

**Affiliations:** 1 Department of Zoology, University of Oxford, Oxford, UK.; 2 Instituto de Medicina Tropical, Faculdade de Medicina da Universidade de São Paulo, São Paulo, Brazil.; 3 Departamento de Moléstias Infecciosas e Parasitárias, Faculdade de Medicina da Universidade de São Paulo, São Paulo, Brazil.; 4 Centro de Pesquisa em Virologia, Faculdade de Medicina de Ribeirão Preto, Ribeirão Preto, Brazil.; 5 Departamento de Genética, Instituto de Biologia, Universidade Federal do Rio de Janeiro, Rio de Janeiro, Brazil.; 6 Spatial Epidemiology Lab, Université Libre de Bruxelles, Brussels, Belgium.; 7 Department of Microbiology, Immunology and Transplantation, Rega Institute, KU Leuven, Leuven, Belgium.; 8 MRC Centre for Global Infectious Disease Analysis, J-IDEA, Imperial College London, London, UK.; 9 Institute for Applied Economic Research, Brasília, Brazil.; 10 Université Clermont Auvergne, INRAE, VetAgro Sup, UMR EPIA, Saint-Genès-Champanelle, France.; 11 Laboratório de Bioinformática, Laboratório Nacional de Computação Científica, Petrópolis, Brazil.; 12 Departamento de Genética, Evolução, Microbiologia e Imunologia, Instituto de Biologia and Experimental Medicine Research Cluster (EMRC), Universidade Estadual de Campinas, Campinas, Brazil.; 13 Department of Mathematics, Imperial College London, London, UK.; 14 Escola de Matemática Aplicada (EMAp), Fundação Getúlio Vargas, Rio de Janeiro, Brazil.; 15 Department of Electronic Systems Engineering, University of São Paulo, São Paulo, Brazil.; 16 Usher Institute, University of Edinburgh, Edinburgh, UK.; 17 Department of Clinical and Toxicological Analyses, School of Pharmaceutical Sciences, University of São Paulo, São Paulo, Brazil.; 18 Departamento de Matemática Aplicada, Instituto de Matemática e Estatística, Universidade de São Paulo, São Paulo, Brazil.; 19 Department of Infectious Disease Epidemiology, Faculty of Epidemiology and Population Health, London School of Hygiene & Tropical Medicine, London, UK.; 20 Centre for the Mathematical Modelling of Infectious Diseases, London School of Hygiene & Tropical Medicine, London, UK.; 21 Institute for Microbiology and Infection, University of Birmingham, Birmingham, UK.; 22 DB Diagnósticos do Brasil, São Paulo, Brazil.; 23 LIM 03 Laboratório de Medicina Laboratorial, Hospital das Clínicas Faculdade de Medicina da Universidade de São Paulo, São Paulo, Brazil.; 24 Instituto Hermes Pardini, Belo Horizonte, Brazil.; 25 Departamento de Cirurgia, Faculdade de Medicina, Universidade Federal de Minas Gerais, Belo Horizonte, Brazil.; 26 Simile Instituto de Imunologia Aplicada Ltda, Belo Horizonte, Brazil.; 27 Laboratório DASA, São Paulo, Brazil.; 28 Laboratório Fleury, São Paulo, Brazil.; 29 Laboratório de Virologia, Instituto de Ciências Biomédicas, Universidade Federal de Uberlândia, Uberlândia, Brazil.; 30 Centro de Estudos da Biodiversidade, Universidade Federal de Roraima, Boa Vista, Brazil.; 31 Divisão de Doenças Infecciosas, Faculdade de Ciências Médicas, Universidade Estadual de Campinas, Campinas, Brazil.; 32 Hospital Estadual Sumaré, Universidade Estadual de Campinas, Campinas, Brazil.; 33 Departamento de Doenças Infecciosas e Parasitárias, Faculdade de Medicina, Universidade Federal do Rio de Janeiro, Rio de Janeiro, Brazil.; 34 Divisão de Laboratório Central do Hospital das Clínicas, da Faculdade de Medicina da Universidade de São Paulo, São Paulo, Brazil.; 35 Department of Pathobiology and Population Sciences, Royal Veterinary College, Hatfield, UK.; 36 University of Oxford, Latin American Centre, Oxford School of Global and Area Studies, Oxford, UK.; 37 Departamento de Clínica Médica, Faculdade de Ciências Médicas, Universidade Estadual de Campinas, Campinas, Brazil.; 38 Departamento de Patologia Clínica, Faculdade de Ciências Médicas, Universidade Estadual de Campinas, Campinas, Brazil.; 39 Departamento de Genética, Ecologia e Evolução, Instituto de Ciências Biológicas, Universidade Federal de Minas Gerais, Belo Horizonte, Brazil.; 40 Departamento de Botânica, Instituto de Ciências Biológicas, Universidade Federal de Minas Gerais, Belo Horizonte, Brazil.; 41 Departamento de Bioquímica e Imunologia, Universidade Federal de Minas Gerais, Belo Horizonte, Brazil.; 42 Departamento de Biologia Celular, Instituto de Ciências Biológicas, Universidade Federal de Minas Gerais, Belo Horizonte, Brazil.; 43 Centro de Saúde da Comunidade, Universidade Estadual de Campinas, Campinas, Brazil.; 44 Centro de Laboratórios Multiusuários, Instituto de Ciências Biológicas, Universidade Federal de Minas Gerais, Belo Horizonte, Brazil.; 45 Laboratório de Pesquisas em Virologia, Faculdade de Medicina de São José do Rio Preto, São José do Rio Preto, São Paulo, Brazil.; 46 Mathematical Sciences, University of Southampton, Southampton, UK.; 47 Institute of Evolutionary Biology, University of Edinburgh, Edinburgh, UK.

## Abstract

Brazil currently has one of the fastest growing SARS-CoV-2 epidemics in the world. Owing to limited available data, assessments of the impact of non-pharmaceutical interventions (NPIs) on virus spread remain challenging. Using a mobility-driven transmission model, we show that NPIs reduced the reproduction number from >3 to 1–1.6 in São Paulo and Rio de Janeiro. Sequencing of 427 new genomes and analysis of a geographically representative genomic dataset identified >100 international virus introductions in Brazil. We estimate that most (76%) of the Brazilian strains fell in three clades that were introduced from Europe between 22 February11 March 2020. During the early epidemic phase, we found that SARS-CoV-2 spread mostly locally and within-state borders. After this period, despite sharp decreases in air travel, we estimated multiple exportations from large urban centers that coincided with a 25% increase in average travelled distances in national flights. This study sheds new light on the epidemic transmission and evolutionary trajectories of SARS-CoV-2 lineages in Brazil, and provide evidence that current interventions remain insufficient to keep virus transmission under control in the country.

Severe acute respiratory syndrome coronavirus 2 (SARS-CoV-2) is a novel betacoronavirus with a 30-kb genome that was first reported in December 2019 in Wuhan, China (*1*, *2*). SARS-CoV-2 was declared a public health emergency of international concern on 30 January 2020. As of 12 July 2020, coronavirus disease 2019 (COVID-19) has caused over 12.5 million cases and 561 thousand deaths globally (*3*). The virus can be classified into two main phylogenetic lineages, namely A and B, that spread from Wuhan before strict travel restrictions were enacted (*4*, *5*) and now co-circulate around the world (*6*). The case fatality ratio of SARS-CoV-2 infection has been estimated between 1.2 and 1.6% (*7*–*9*) with substantially higher ratios in those aged above 60 years (*8*). Some estimates suggest that 18-56% of SARS-CoV-2 transmission is from asymptomatic or pre-symptomatic individuals (*10*–*13*), complicating epidemiological assessments and public health efforts to curb the pandemic.

## Challenges of real-time assessment of transmission

**Fig. 1:**
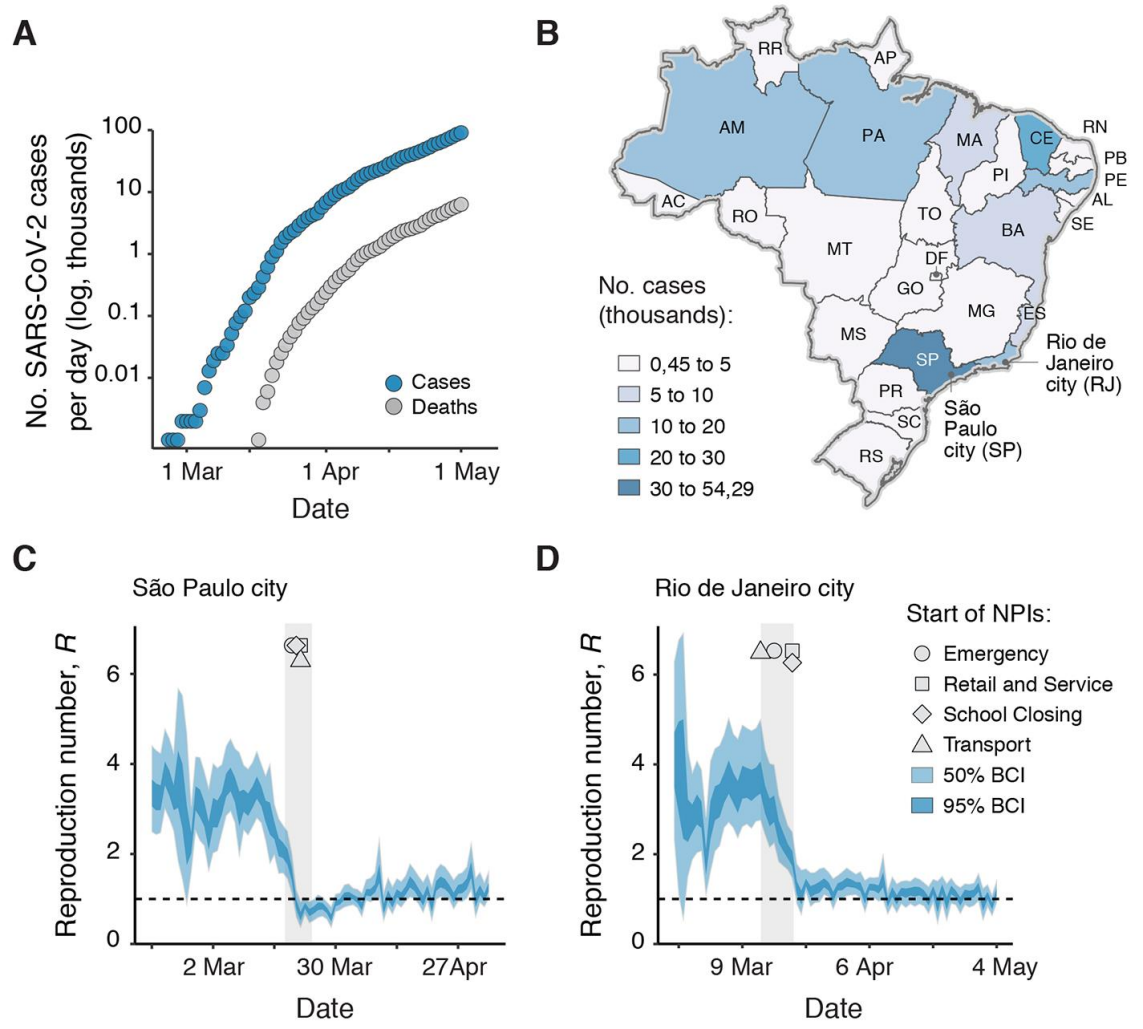
SARS-CoV-2 epidemiology and epidemic spread in Brazil. (**A**) Cumulative number of SARS-CoV-2 reported cases (blue) and deaths (grey) in Brazil. (**B**) States are colored according to the number of cumulative confirmed cases by 30 April 2020. (**C** and **D**) Reproduction number (*R*) over time for the cities of São Paulo (C) and Rio de Janeiro (D). *R* were estimated using a Bayesian approach incorporating daily number of deaths and four variables related to mobility data (a social isolation index from Brazilian geolocation company *InLoco*, and Google mobility indices for time spent in transit stations, parks, and the average between groceries and pharmacies, retail and recreational, and workspaces). Dashed horizontal line indicates *R* = 1. Grey area and geometric symbols show the times at which NPIs interventions were implemented. Bayesian credible intervals (BCIs, 50 and 95%) are shown as shaded areas. The 2-letter ISO 3166-1 codes for the 27 federal units in Brazil are provided in Supplementary Information.

While the SARS-CoV-2 epidemics in several countries including China, Italy, and Spain have been brought under control through non-pharmaceutical interventions (NPIs) (*3*), the number of SARS-CoV-2 cases and deaths in Brazil continues to increase (*14*) (Fig. 1A). As of 12 July 2020, Brazil has now reported 1,800,827 SARS-CoV-2 cases, the second largest number in the world, and 70,398 deaths. Over a third of the cases (34%) in Brazil are concentrated in the southeast region which includes São Paulo city (Fig. 1B), the world’s fourth largest conurbation, where the first case in Latin America was reported on 25 February 2020 (*15*). Diagnostic assays for SARS-CoV-2 molecular detection were widely distributed across the regional reference centres of the national public health laboratory network from 21 February 2020 onwards (*16*, *17*). However, several factors, including delays in reporting, changes in notification, and heterogeneous access to testing across populations, obfuscate the real-time assessment of virus transmission using SARS-CoV-2 case counts (*15*). Consequently, a more accurate measure of SARS-CoV-2 transmission in Brazil is provided by reported deaths due to severe acute respiratory infections (SARI), provided by the Sistema Único de Saúde (SUS) (*18*). Changes in the opportunity for SARS-CoV-2 transmission are strongly associated with changes in average mobility (*18**–**20*), and can typically be measured by calculating the effective reproduction number, *R,* defined as the average number of secondary infections caused by an infected person. *R* >1 indicates a growing epidemic while *R* <1 is needed to achieve a decrease in transmission.

We used a Bayesian semi-mechanistic model (*21*, *22*) to analyze SARI mortality statistics and human mobility data to estimate daily changes in *R* in São Paulo city (12,2 million inhabitants) and Rio de Janeiro city (6,7 million inhabitants), the largest urban metropoles in Brazil (Fig. 1, C and D). NPIs in Brazil consisted of school closures implemented between 12 and 23 March 2020 across the country’s 27 federal units/states, and store closures implemented between 13 and 23 March 2020. In São Paulo city, schools started closing on 16 March and stores closed four days later. At the start of the epidemics, we found *R >3* in São Paulo and Rio de Janeiro, and that concurrent with the timing of state mandated NPIs, *R* values fell close to 1.

## Mobility driven reproduction number changes

Analysis of the reproduction number after NPI implementation highlights several notable mobility-driven features. There was a period immediately following NPIs, between 21 and 31 March 2020, when *R* was consistently <1 in São Paulo city (Fig. 1C). However, after this initial decrease, the *R* value for São Paulo rises >1 and increases through time, a trend associated with increased population mobility. This can be seen in the Google transit stations index, which rises from -60% to -52%, and by a decrease in the social isolation index from 54% to 47%. By 4 May 2020, we estimate *R* = 1.3 (BCI 95%: 1.0-1.6) in both São Paulo and Rio de Janeiro cities (table S1). However, we note that there were instances in the previous 7 days when the 95% credible intervals for *R* included values below 1, drawing attention to the fluctuations and uncertainty in the estimated reproduction number for both cities.

Early sharing of genomic sequences, including the first SARS-CoV-2 genome, Wuhan-Hu-1, released on 10 January (*23*), has enabled unprecedented global levels of molecular testing for an emerging virus (*24*, *25*). However, despite the thousands of virus genomes deposited on public access databases, there is a lack of consistent sampling structure, and limited data from Brazil (*26*–*28*), which hampers accurate reconstructions of virus movement and transmission using phylogenetic analyses. To investigate how SARS-CoV-2 became established in the country, and to quantify the impact of NPIs on virus spatiotemporal spread, we tested a total of 26,732 samples from public and private laboratories using real-time PCR assays and found 7,944 (29%) to be positive for SARS-CoV-2. We then focused our sequencing efforts on generating a large and spatially representative genomic dataset with curated metadata in order to maximise the association between the number of sequences and the number of SARS-CoV-2 confirmed cases per state.

## Spatially representative sequencing efforts

**Fig. 2:**
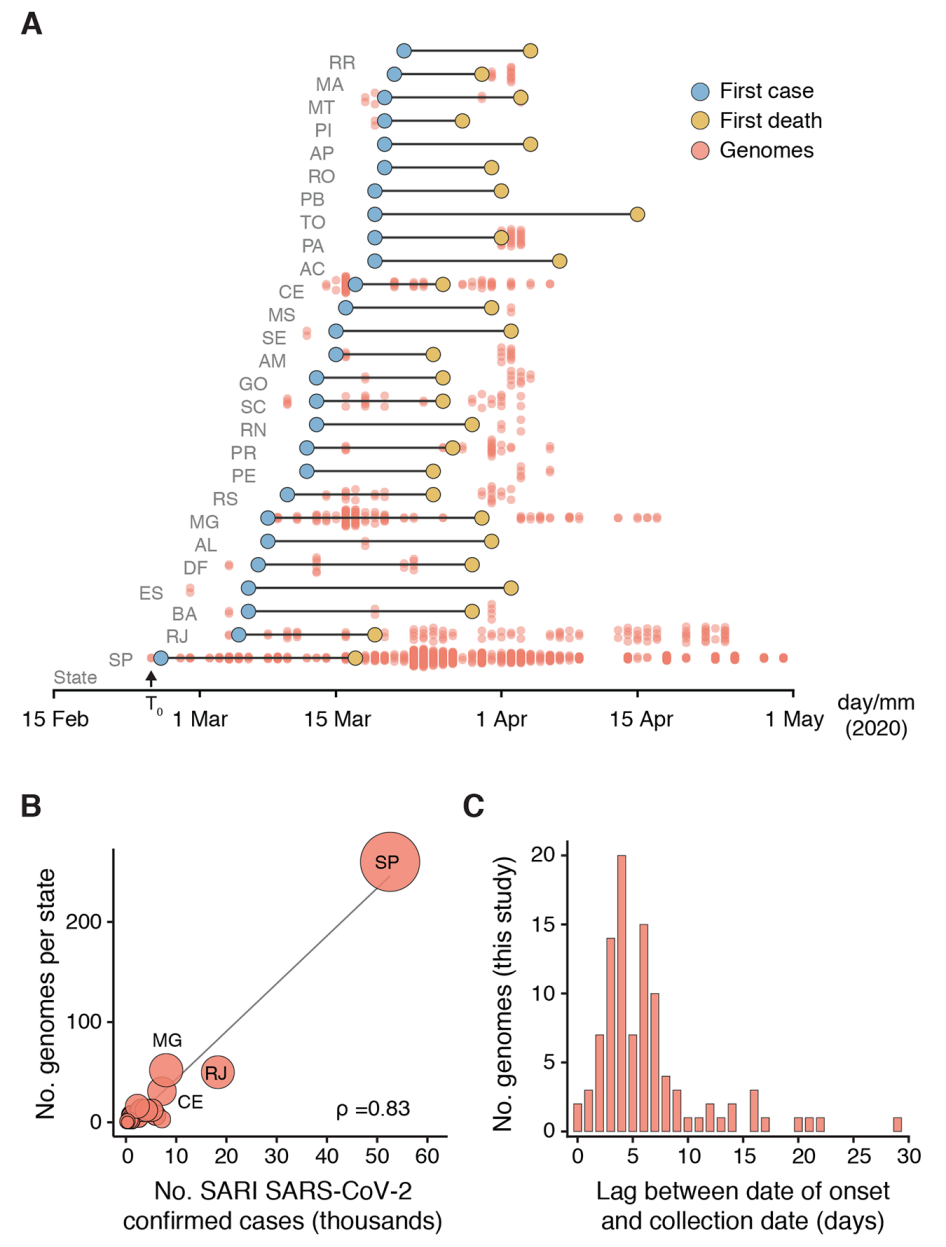
Spatially-representative genomic sampling. (**A**) Dumbbell plot showing the time intervals between date of collection of sampled genomes, notification of first cases and first deaths in each state. Red lines indicate the lag between the date of collection of first genome sequence and first reported case. The key for the 2-letter ISO 3166-1 codes for Brazilian federal units (or states) are provided in Supplementary Information. (**B**) Spearman’s rank (ρ) correlation between the number of SARI SARS-CoV-2 confirmed and SARI cases with unknown aetiology against number of sequences for each of the 21 Brazilian states included in this study (see also fig. S4). Circle sizes are proportional to the number of sequences for each federal unit. (**C**) Interval between the date of symptom onset and date of sample collection for the sequences generated in this study.

We generated 427 new SARS-CoV-2 genomes with >75% genome coverage from Brazilian samples collected between 5 March and 30 April 2020 (figs. S1 to S3 and data S1). For each state, the time between the date of the first reported case and the collection date of the first sequence analyzed in that state was only 4.5 days on average (Fig. 2A). For eight federal states, genomes were obtained from samples collected up to 6 days before the first case notifications. The genomes generated here were collected in 85 municipalities across 18 of 27 federal units spanning all regions in Brazil (Fig. 2A and fig. S2). Sequenced genomes were obtained from samples collected on average 4 days (median, range: 0 to 29 days) after onset of symptoms and were generated in 3 laboratories using harmonized sequencing and bioinformatic protocols (table S2). When we include 63 additional available sequences from Brazil deposited in GISAID (29) (see data S1 and S2), we find the data set is representative of the spatial heterogeneity of the Brazilian epidemic. Specifically, the number of genomes per state strongly correlates with SARI SARS-CoV-2 confirmed cases and SARI cases with unknown aetiology per state (*n* = 490 sequences from 21 states, Spearman’s correlation, ρ = 0.83; Fig. 2A). This correlation varied from 0.70 to 0.83 when considering SARI cases and deaths caused by SARS-CoV-2, and SARI cases and deaths from unknown aetiology (fig. S4). Most (*n* = 485/490) Brazilian sequences belong to SARS-CoV-2 lineage B, with only 5 strains belonging to lineage A (2 from Amazonas, 1 from Rio Grande do Sul, 1 from Minas Gerais and 1 from Rio de Janeiro; data S1 and fig. S5 show detailed lineage information for each sequence). Moreover, we used an *in-silico* assessment of diagnostic assay specificity for Brazilian strains (*n = *490) to identify potential mismatches in some assays targeting Brazilian strains. We find that the forward primers of the Chinese CDC and Hong Kong University nucleoprotein-targeting RT-qPCR may be less appropriate for use in Brazil than other diagnostic assays, for which few or no mismatches were identified (fig. S6 and table S3). The impact of these mismatches on the sensitivity of these assays should be confirmed experimentally. If sensitivity is affected, the use of duplex RT-qPCR assays that concurrently target different genomic regions may help the detection of viruses with variants in primer or probe binding regions.

## Phylogenetic analyses and international introductions

**Fig. 3:**
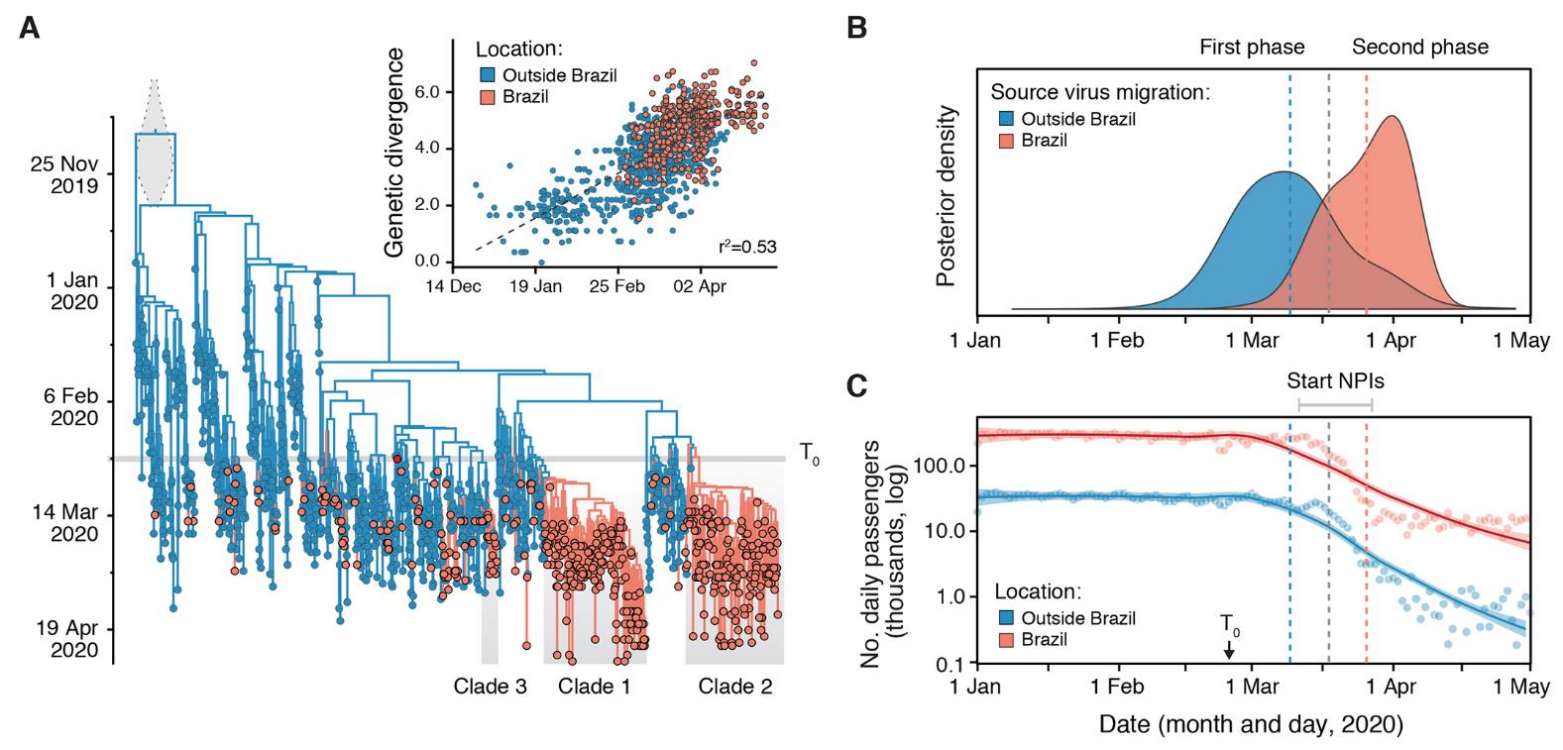
Evolution and spread of SARS-CoV-2 in Brazil. (**A**) Time-resolved maximum clade credibility phylogeny of 1182 SARS-CoV-2 sequences, 490 from Brazil (red) and 692 from outside Brazil (blue). The largest Brazilian clades are highlighted by grey boxes (*Clade 1*, *Clade 2* and *Clade 3*). The panel A inset shows a root-to-tip regression of genetic divergence against dates of sample collection. (**B**) Dynamics of SARS-CoV-2 import events in Brazil. Dates of international and national (between federal states) migration events were estimated from virus genomes using a phylogeographic approach. The first phase was dominated by virus migrations from outside Brazil while the second phase is marked by virus spread within Brazil. Dashed vertical lines correspond to the mean posterior estimate for migration events from outside Brazil (blue) and within Brazil (red). (**C**) Locally estimated scatterplot smoothing of the daily number of international (blue) and national (red) air passengers in Brazil in 2020. T_0_ = date of first reported case in Brazil (25 February 2020).

We estimated maximum likelihood and molecular clock phylogenies for a global dataset with a total of 1,182 genomes sampled from 24 Dec 2019 to 30 Apr 2020 (root-to-tip genetic distance correlation with sampling dates, r^2^ = 0.53; Fig. 3A and fig. S7). We inferred a median evolutionary rate of 1.13 × 10^−3^ (95% BCI: 1.03–1.23 × 10^−3^) substitutions per site per year (s/s/y), using an exponential growth coalescent model, equating to 33 changes per year on average across the virus genome. This is within the range of evolutionary rates estimated for other human coronaviruses (*30*–*33*). We estimate the date of the common ancestor (TMRCA) of the SARS-CoV-2 pandemic to around mid-Nov 2019 (median = 19 Nov 2019, 95% BCI: 26 Oct 2019 to 6 Dec 2019), in line with recent findings (*34*, *35*).

Phylogenetic analysis revealed that the majority of the Brazilian genomes (76%, *n* = 370/490) fell into three clades hereafter named as Clade 1 (*n* = 186/490, 38% of Brazilian strains), Clade 2 (*n* = 166, 34%) and Clade 3 (*n* = 18/490, 4%; Fig. 3A and figs. S8 and S9), which were largely in agreement with those identified in a phylogenetic analysis using 13,833 global genomes. The most recent common ancestors of the three main Brazilian clades (Clades 1 to 3) were dated from 28 February (21 Feb to 4 Mar 2020) (*Clade 1*), 22 February (17 to 24 Feb 2020) (Clade 2) to 11 March (9 to 12 Mar 2020) (Clade 3) (Fig. 3A and fig. S10). This indicates that community-driven transmission was already established in Brazil by early March, suggesting that international travel restrictions initiated after this period would have had limited impact. Brazilian Clade 1 is characterized by a nucleotide substitution in the spike protein (G25088T, numbering relative to GenBank reference NC_045512.2) and circulates predominantly in São Paulo state (*n* = 159, 85.4%; figs. S9 and S11). Clade 2 is defined by two nucleotide substitutions in ORF6 (T27299C) and nucleoprotein (T29148C); this is the most spatially widespread lineage, with sequences from a total of 16 states in Brazil. Clade *3* is concentrated in Ceará state (*n* = 16, 89%) and falls in a global cluster with sequences mainly from Europe. In the Amazon region, where the epidemic is expanding rapidly (*14**, **22*), we find evidence for multiple national and international introductions, with 37% (*n* = 7/19) of sequences from Pará and Amazonas states clustering in Clade 1 and 32% (*n* = 6/19) in Clade 2.

Time-measured phylogeographic analyses revealed at least 102 (95% BCI: 95–109) international introductions of SARS-CoV-2 in Brazil (Fig. 3A and figs. S8 and S12). This represents an underestimate of the real number of introductions, as we have sequenced, on average, only 1 out of 200 confirmed cases. Most of these estimated introductions were directed to internationally well-connected states (*36*) such as São Paulo (36% of all imports), Minas Gerais (24%), Ceará (10%) and Rio de Janeiro (8%) (fig. S12). We further assessed the contribution of international vs. national virus lineage movement events through time (Fig. 3B). In the first phase of the epidemic, we find an increasing number of international introductions until 10 Mar 2020 (Fig. 2B). Limited available travel history data (*15*) suggests that these early cases were predominantly acquired from Italy (26%, *n* = 70 of 266 unambiguously identified country of infection) and the USA (28%, *n* = 76 of 266). After this initial phase, we find that the estimated number of international imports decreased concomitantly with the decline in the number of international passengers travelling to Brazil (Fig. 3, B and C, and S13). In contrast, despite the declines in the number of passengers travelling on national flights (Fig. 3C), we detected an increase in virus lineage movement events between Brazilian regions at least until early April 2020.

## Modelling spatiotemporal spread within Brazil

**Fig. 4:**
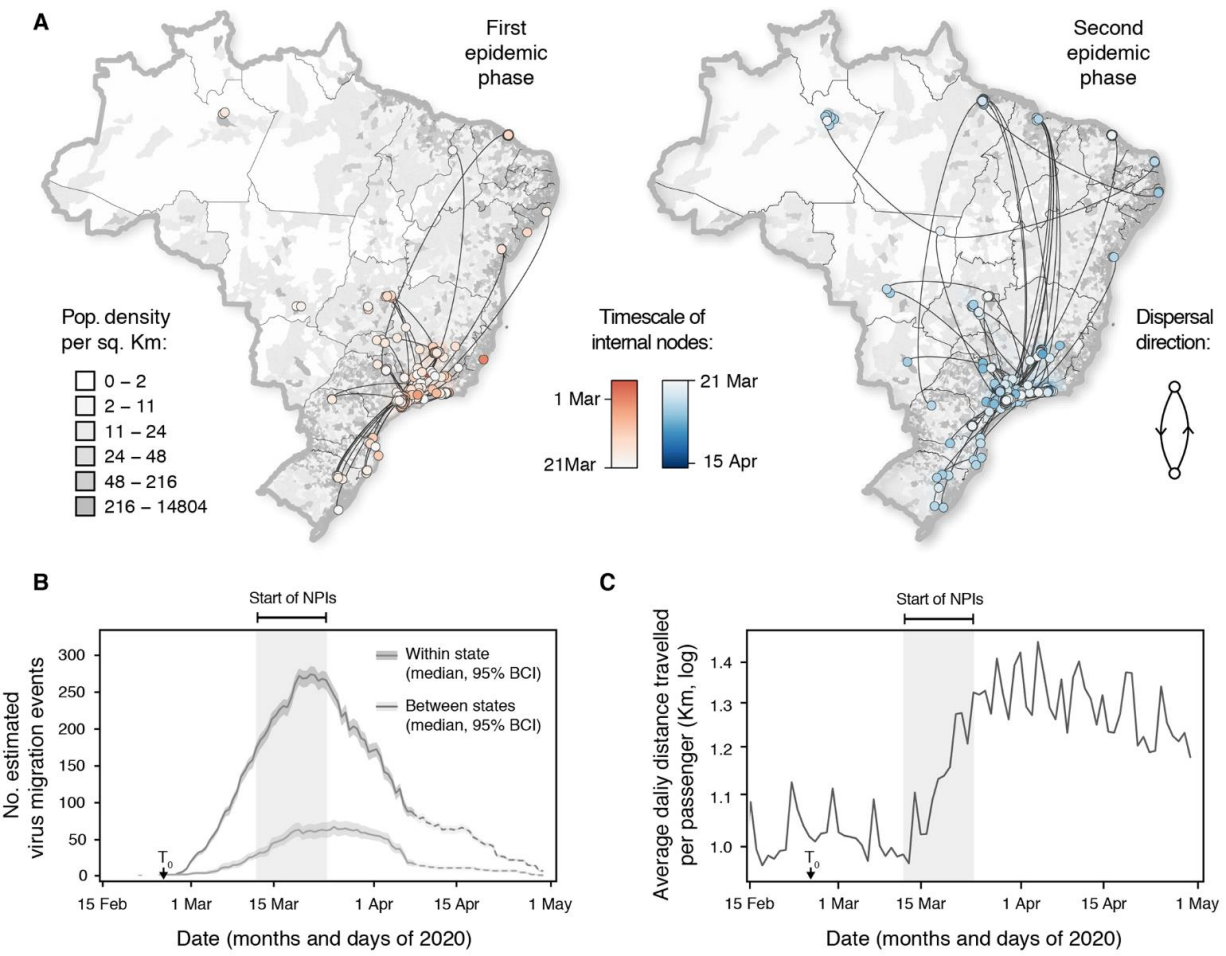
Spread of SARS-CoV-2 in Brazil. (**A**) Spatiotemporal reconstruction of the spread of Brazilian SARS-CoV-2 clusters containing >2 sequences during the first (left) and the second epidemic phase (right) epidemic phase (Fig. 3B). Circles represent nodes of the MCC phylogeny and are colored according to their inferred time of occurrence. Shaded areas represent the 80% highest posterior density (HPD) interval and depict the uncertainty of the phylogeographic estimates for each node. Solid curved lines denote the links between nodes and the directionality of movement. Sequences belonging to clusters with <3 sequences were also plotted on the map with no lines connecting them. Background population density for each municipality was obtained from the Brazilian Institute of Geography (https://www.ibge.gov.br/). See fig. S14 for details of virus spread in the Southeast region. (**B**) Estimated number of within state (or within a given federal unit) and between-state (or between federal units) virus migrations over time. Dashed lines indicate estimates obtained during period of limited sampling (fig. S2). (**C**) Average distance in kilometres travelled by an air passenger per day in Brazil. Number of daily air passengers is shown in Fig. 3B. Light grey boxes indicate starting dates of NPIs across Brazil.

To better understand virus spread across spatiotemporal scales within Brazil, we use a continuous phylogeographic model that maps phylogenetic nodes to their inferred origin locations (*37*) (Fig. 4). We distinguish branches that remain within a state versus those that cross a state to infer the proportion of within versus between state observed virus movement.

We estimate that during the first epidemic phase, SARS-CoV-2 spread mostly locally and within-state borders. In contrast, the second phase was characterized by long-distance movement events and the ignition of the epidemic outside the southeast region of Brazil (Fig. 4A). Throughout the epidemic, we find that within-state virus lineage movement was, on average, 5.1-fold more frequent than between-state movement. Moreover, our data suggests that within-state virus spread, and to a lesser extent, between-state virus spread, decreased after the implementation of NPIs (Fig. 4B). However, it is useful to note that the more limited sampling after April 6 2020 (see fig. S2) decreases inferred virus lineage movement toward present (Figs. 3B and 4B).

Interestingly, we find that the average route length travelled by passenger increased by 25% during the second phase of the epidemic (Fig. 4C), despite a concomitant reduction in the number of passengers flying within Brazil (Fig. 3C). The increase in the average route length post-NPI implementation results from a larger reduction in the number of air passengers flying on shorter distance journeys compared to those flying longer distance journeys. For example, we find an 8.8-fold reduction in the number of passengers flying in flight legs < 1000 km, compared to a 4.4-fold reduction in those flying >2000 km (fig. S15). These findings emphasize the roles of within and between-state mobility as a key driver of both local and inter-regional virus spread, with highly populated and well-connected urban conurbations in the southeast region acting as main sources of virus exports within the country (fig. S12).

## Discussion

We provide a comprehensive analysis of SARS-CoV-2 spread in Brazil that shows the importance of community and nation-wide measures to control the COVID-19 epidemic Brazil. Although NPIs initially reduced virus transmission and spread, the continued increase in the number of cases and deaths in Brazil highlights the urgent need to prevent future virus transmission by implementing rapid and accessible diagnostic screening, contact tracing, quarantining of new cases and coordinated social and physical distancing measures across the country (*38*). With the recent relaxation of NPIs in Brazil and elsewhere, continued molecular, immunological and genomic surveillance are required for real-time data-driven decisions. Our analysis shows how changes in mobility may impact global and local transmission of SARS-CoV-2, and demonstrates how combining genomic and mobility data can complement traditional surveillance approaches.

## Supplementary Materials

science.sciencemag.org/cgi/content/full/science.abd2161/DC1 Materials and Methods Figs. S1 to S15 Tables S1 to S3 List of Members of the CADDE Genomic Network References (*41*–*77*) Data S1 and S2 MDAR Reproducibility Checklist
